# Natural collagen scaffold with intrinsic piezoelectricity for enhanced bone regeneration

**DOI:** 10.1016/j.mtbio.2025.101532

**Published:** 2025-01-29

**Authors:** Jing Han, Zhao Li, Jing Du, Qun Zhang, Shaohua Ge, Hong Liu, Baojin Ma

**Affiliations:** a Department of Tissue Engineering & Periodontology, School and Hospital of Stomatology, Shandong University & Shandong Key Laboratory of Oral Tissue Regeneration & Shandong Engineering Research Center of Dental Materials and Oral Tissue Regeneration & Shandong Provincial Clinical Research Center for Oral Diseases, Jinan, Shandong, 250012, China; b College of Materials Science and Engineering, Qingdao University of Science & Technology, Qingdao, Shandong, 266061, China; c State Key Laboratory of Crystal Materials, Shandong University, Jinan, Shandong, 250013, China

**Keywords:** Natural collagen, Intrinsic piezoelectricity, Osteogenesis, PI3K-Akt signaling pathway, Bone tissue regeneration

## Abstract

Materials-mediated piezoelectric signals have been widely applied in bone regeneration. Collagen is the most abundant protein in the human body, and native collagen with complete tertiary structure shows efficient piezoelectricity. However, the traditional collagen scaffolds are lack of piezoelectricity due to the destruction of the complete tertiary structure. Here, natural collagen scaffolds with the complete tertiary structure were prepared. Alkali treatment made the collagen scaffold lose piezoelectricity. The collagen with/without piezoelectricity (PiezoCol/NCol) scaffolds both possessed good cytocompatibility and promoted cell adhesion. After being implanted subcutaneously, the NCol scaffold almost did not affect bone regeneration with/without ultrasound treatment. However, under ultrasound treatment, the PiezoCol scaffold promoted the new bone formation with enhanced osteogenic differentiation, angiogenesis, and neural differentiation, meaning that piezoelectricity endows collagen with satisfactory promotion for bone regeneration. Meanwhile, the PiezoCol scaffold can also accelerate bone formation without ultrasound treatment, which should be attributed to the daily exercise-caused weak piezoelectric stimulation. Further, the proteomic analysis revealed the mechanism by which the PiezoCol scaffold promoted bone tissue formation via mainly upregulating the PI3K-Akt signaling pathway. This study provides a new strategy to enhance the osteoinduction of collagen scaffold for bone regeneration by maintaining intrinsic piezoelectricity.

## Highlights

• Natural collagen scaffolds with the complete tertiary structure have been prepared.
• The collagen with/without piezoelectricity (PiezoCol/NCol) scaffolds possessed good cytocompatibility and promoted cell adhesion.
• The PiezoCol scaffold promoted the new bone formation with enhanced osteogenic differentiation, angiogenesis, and neural differentiation.

## Graphical abstract

**Figure:**
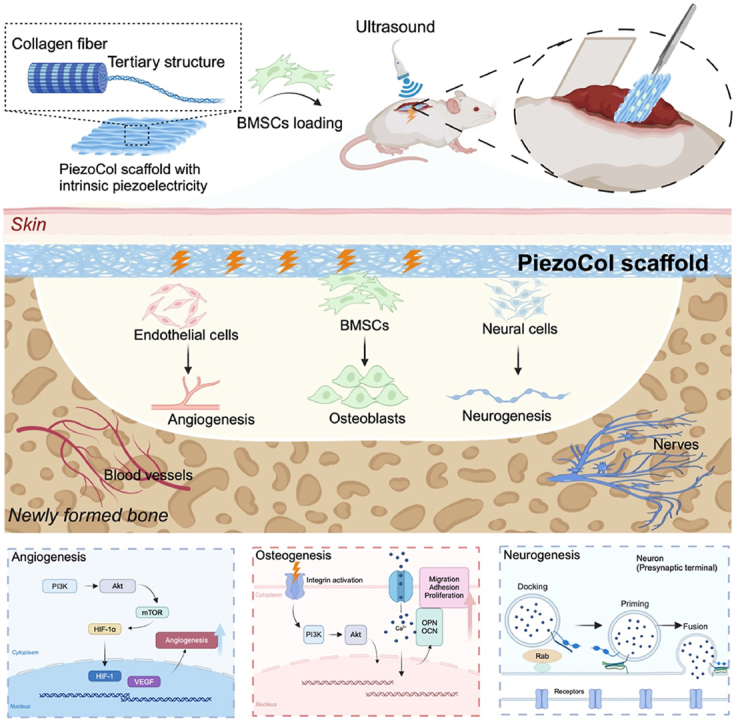

## 1 Introduction

Bone defects caused by various diseases such as tumors, osteoporosis, and infection are currently one of the principal causes of disability, which severely compromise life quality and bring a huge burden on social welfare [1]. Traditional treatments for bone defects include autologous or allogenic graft implantation and growth factor loading, which have some drawbacks, such as limited resources, the risk of secondary injury and infection, as well as immunological rejection [2]. Materials-mediated physical signals have been widely applied in tissue engineering due to their high security, application convenience, and low cost, and are regarded as alternatives for growth factors [3]. Among them, the piezoelectric signal shows great potential in regulating cell differentiation and tissue regeneration. For example, a piezoelectric bone cement comprising poly methyl methacrylate and BaTiO_3_ was prepared to promote osteogenic differentiation of stem cells [4], and hydroxyapatite (HAp)/polyvinylidene fluoride-co-trifluoro ethylene P(VDF-TrFE) composite scaffold was used to accelerate bone regeneration through piezoelectricity of P(VDF-TrFE) and inherent osteogenic ability of HAp [5]. Those materials showed potential for bone regeneration, whereas biodegradation and biosecurity are big concerns when applying them *in vivo*. Although degradable piezoelectric polymers like poly-L-lactic acid (PLLA) were developed for tissue regeneration [6], the degradation product of PLLA, lactic acid, is a relatively strong acid and can cause a chronic inflammatory response to retard tissue repair [7]. Additionally, processing costs and time also need to be considered for the feasibility of practical applications. Therefore, constructing natural piezoelectric materials with good biocompatibility, biodegradation, free inflammation, and cost-effectiveness for enhanced bone regeneration is still challenging.

Collagen is the most abundant protein in the human body and has become one of the most important natural and FDA-proved biomaterials for tissue regeneration due to its excellent biocompatibility [8]. Natural collagen possesses a hierarchical self-assembly complex structure. The hierarchical structure includes primary structure (polypeptides), secondary structure (α-helix chains), and tertiary structure (canonical triple helix fiber) [9]. The complete tertiary structure endows collagen with excellent piezoelectricity [10]. Unfortunately, during the preparation process, collagen-based scaffolds usually lose intrinsic piezoelectricity due to the destruction of the tertiary structure [9]. The collagen scaffolds without piezoelectricity almost fail to promote osteogenic differentiation, thereby needing the assistance of growth factors to accelerate bone regeneration. So far, no study has been performed to directly use the intrinsic piezoelectricity of natural collagen for bone repair. Therefore, developing a strategy to maintain the complete tertiary structure would further enhance the biological activity of collagen scaffolds and broaden their application in bone tissue engineering.

**Fig. 1:**
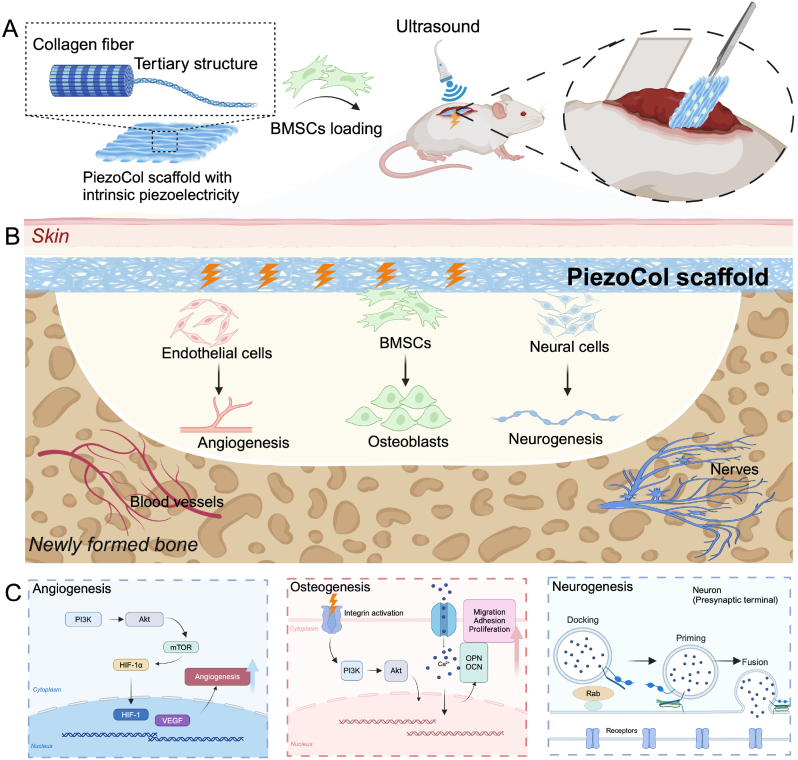
(A, B) Schematic graph of the *in vivo* application of PiezoCol scaffold; (C) The mechanism of the PiezoCol scaffold for enhanced ectopic bone regeneration.

In this study, we proposed a bone regeneration strategy based on natural collagen by applying its intrinsic piezoelectricity. A natural collagen scaffold (PiezoCol) with the complete tertiary structure was first prepared, derived from the porcine acellular dermal matrix. After the strong alkali treatment to destroy the tertiary structure, a denatured collagen scaffold (NCol) without piezoelectricity was obtained. *In vitro* results demonstrated that both collagen scaffolds supported the growth of bone marrow-derived mesenchymal stem cells (BMSCs) with fine attachment and typical morphology. Subsequently, the collagen scaffolds were laden with BMSCs and implanted subcutaneously in the dorsal part of rats for *in vivo* bone regeneration assessment (Fig. 1A). The PiezoCol group under ultrasound treatment showed obvious bone-liked tissue formation after 4 weeks (Fig. 1B). Meanwhile, the piezoelectric signal-induced angiogenesis and neurogenesis were also beneficial for bone regeneration [11]. The proteomic analysis further revealed that the PiezoCol scaffold supported bone-like tissue formation via upregulating the Integrin/PI3K-Akt signaling axis (Fig. 1C). Furthermore, the calcium signaling pathway also participated in regulating calcium ossification. By the synergistic effect of osteogenesis, angiogenesis, and neurogenesis, the PiezoCol scaffold can efficiently enhance bone regeneration stimulated by the intrinsic piezoelectric signal under non-invasive ultrasonic treatment, which provides a new strategy for bone defect treatment.

## 2 Experimental section

### 2.1 Scaffold fabrication

All the chemicals were purchased from Aladdin if there is no explanation. PiezoCol scaffold was prepared according to our previous report [12]. The fresh full-thickness porcine skin was purchased from a local market after removing hair and subdermal fat tissue. To obtain decellularized PiezoCol, the skin was treated using the following steps: (1) 2.0 g/L NaOH and 0.8 g/L sodium dodecyl sulfate (SDS) mixed solution for 12 h; (2) deionized water for 15 min, 2 times; (3) 2.4 g/L SDS for 2 h; (4) deionized water for 15 min, 2 times; (5) 3.0 g/L pancreatin for 12 h; (6) deionized water for 15 min, 2 times; (6) 2.4 g/L SDS for 2 h; (7) deionized water for 15 min, 2 times; (7) 6 % NaCl for 6 h; (8) deionized water for 20 min, 3 times. To obtain NCol, PiezoCol was immersed in 1 M NaOH for 30 min. After freeze-drying, PiezoCol and NCol were cut into square membranous scaffolds with a length of about 1 cm and a thickness of about 1 mm.

### 2.2 Characterization of PiezoCol and NCol scaffolds

The morphology of the PiezoCol and NCol scaffold was observed by a scanning electron microscope (SEM, Hitachi, S-4800). X-ray powder diffraction (XRD) patterns were recorded by an X-ray diffractometer (Bruker D8 Advance, Germany, Cu Kα). Fourier-transform infrared spectroscopy (FTIR) spectra were recorded for PiezoCol and NCol by an IR spectrophotometer (Thermo Nicolet, NEXUS 670). Differential scanning calorimetry (DSC) measurements were performed with a DSC3 (Mettler Toledo, Switzerland) under a nitrogen atmosphere. About 10 mg of scaffolds were subjected to the whole DSC protocol, in which both samples were heated to 100 °C at a rate of 10 °C/min. The mechanical performances of the scaffolds were evaluated by tensile tests. The tensile properties of scaffolds (5 × 20 mm^2^) were tested by the universal testing machine (Instron 3340, USA). The degradation behavior of PiezoCol and NCol scaffolds was evaluated by immersing the specimens in phosphate-buffered saline (PBS) containing collagenase (0.5 mg/mL) in a shaking bath maintained at 37 °C. The solution was replaced every two days to preserve the degraded environment. At predetermined time intervals (days 0, 1, 3, 5, and 7), the scaffolds were retrieved, washed to remove residual PBS, lyophilized, and weighed. The weight loss ratio was calculated using the following equation:

Weight loss ratio (%) = (W1 – W2) / W1 × 100

where W1 and W2 denote the initial and final weights of the specimens, respectively. The piezoelectric properties of PiezoCol and NCol were measured by piezo-response force microscopy (PFM, Bruker Dimension Icon Scanning Probe Microscope with a SCM-PIT, Pt-coated conductive tip, with the contact mode, Germany).

### 2.3 Cell culture

The research protocol was approved by the Ethics Committee of the Stomatological Hospital of Shandong University (Protocol Number: 20230809). According to the previous method, four-week-old Wistar rats (80–120 g, male) were used to extract rat BMSCs. Briefly, after being anesthetized and executed, the rats were sterilized with 75 % ethanol. The femur and tibia were isolated independently, and the bone end was cut off to expose the bone marrow which was flushed by Dulbecco's modified Eagle medium (DMEM, Gibco, America) medium containing 20 % fetal bovine serum (FBS, Bio-Ind, Israel) and 1 % penicillin-streptomycin (Biosharp, China). Cells in passages 3–6 were used for the following research. The passaged BMSCs were cultured in DMEM containing 10 % FBS and 1 % penicillin-streptomycin. The neuroendocrine cell line PC12 cells derived from rat pheochromocytoma were cultured in RPMI medium supplemented with 10 % horse serum, 5 % fetal bovine serum, 2 mM glutamine, and 1 % penicillin and streptomycin. The cells were cultured in a humidified environment containing 5 % CO_2_ at 37 °C, and the culture media were refreshed every 2 days.

### 2.4 Cell viability measurement

Following 24 and 48-h of incubation in a 96-well-plate, BMSCs on scaffolds were washed twice with PBS and then incubated with 100 μL 10 v/v% CCK-8 solution (Solarbio) in the medium for another 2 h according to the manufacturer's guidelines. Subsequently, the optical density (OD) value was spectrophotometrically measured using a microplate reader (Bio-Tek FL600 microplate fluorescence reader, Biotek) at a wavelength of 450 nm (OD450).

### 2.5 Cell morphology observation

To access cell morphology, BMSCs cultured on PiezoCol or NCol scaffold underwent PBS washing and fixation in a 2 wt% glutaraldehyde solution in 0.1 M sodium-cacodylate for 20 min. Subsequently, cells were dehydrated using a series of graded ethanol concentrations (ranging from 50 % to 100 % ethanol, alongside 100 % water-free ethanol) for 5 min each. After dehydration, the samples were air-dried in tetramethylsilane. Then the samples were sputter-coated with gold and imaged by SEM.

### 2.6 Cytoskeleton observation

Following the 48-h culture on PiezoCol or NCol scaffold, BMSCs were fixed in 4 % paraformaldehyde (PFA) for 15 min at room temperature and then permeabilized for 5 min. The cells were washed with PBS and stained by F-actin (Abcam, diluted at 1:200) and DAPI (Sigma, 1:2500) to access their morphologies. Stained samples were observed and imaged using fluorescent microscopy (OLYMPUS IX73).

### 2.7 Intracellular Ca^2+^ measurement

The content of Ca^2+^ in BMSCs with/without piezoelectric stimulation was measured. Briefly, cells were first cultured in differentiation media at 37 °C for 1 week. After PBS washing, differentiated cells were stained with Fluo-3-AM (5 μM) for 20 min according to the manufacturer's instruction, intracellular fluorescence was observed and analyzed through fluorescent microscopy (Olympus IX71). The relative Ca^2+^ content was calculated through fluorescence density.

### 2.8 Ectopic osteogenesis model

To verify the osteogenic effect of the PiezoCol scaffold, ectopic osteogenesis was evaluated using a subcutaneous implantation model in rats. The animal experiment was processed and approved by the Ethics Committee of the Stomatological Hospital of Shandong University, Jinan, China (Protocol Number: 20230809). Before implantation, BMSCs were seeded onto NCol and PiezoCol scaffolds (5 × 5 mm) with 2 × 10^5^ cells per scaffold.

24 Wistar rats (8 weeks, 200–250 g, male, SPF) were randomly divided into 6 groups: Control, NCol, PiezoCol without ultrasound treatment (denoted as Ctrl, NCol, and PiezoCol); Control, NCol, PiezoCol with ultrasound treatment (denoted as Ctrl/U, NCol/U, and PiezoCol/U). Each group contained 4 rats. Isoflurane inhalation anesthesia was performed to anesthetize rats before implantation. The BMSCs-laden scaffolds were implanted subcutaneously in the dorsal part of rats, while the same amount of BMSCs were injected subcutaneously in control groups. 4 repeats of implantation were performed in each rat. After surgery, the ultrasound treatment was performed for 10 min at 0.15 W/cm^2^ (1 MHz) every 2 days [13]. At 4 weeks after implantation, rats were sacrificed, and the specimens of surgical-related areas and major organs of rats were harvested.

### 2.9 Micro-computed tomography (Micro-CT) scanning and analysis

A micro-CT scanner (Quantum GX, PerkinElmer) was utilized for radiological imaging and quantitative analysis of bone formation. The volume and area of the newly formed bone-like tissue were measured. In addition, the value of bone volume/area was calculated to compare the quality of the bone-like tissue among different groups.

### 2.10 Histology, immunohistochemical (IHC), and immunofluorescence (IF) staining

After fixation in 4 % PFA for 10 min, the samples were cryo-sectioned into 10 μm thick frozen slices. Subsequently, the frozen slices were stained with a Hematoxylin and Eosin (H&E) staining kit (Solarbio, G1120), a Masson trichrome staining kit (Solarbio, G1340), and a Tartrate-Resistant Acid Phosphatase (TRAP) staining Kit (Solarbi, G1492). IHC and IF staining were performed using osteopontin (OPN) and osteocalcin (OCN), platelet endothelial cell adhesion molecule-1 (PECAM-1 or CD31), class III beta-tubulin (Tuj1), and calcitonin gene-related peptide (CGRP) primary antibodies. The stained slices were observed and imaged using a light or fluorescent microscope (Olympus IX71). The semiquantitative analysis was performed using ImageJ software (National Institutes of Health).

### 2.11 Proteomic analysis

The newly formed bone-like tissues were frozen at −80 °C. Then, a label-free proteomic analysis of three piezoelectricity-induced samples was conducted by the Majorbio Proteomic Service (Shanghai) according to their instruction. During the Differentially expressed proteins (DEP) analysis, |log2FC| ≥ 1 and *p*-value < 0.05 were used as the screening criterion. Hierarchical clustering analysis was conducted on the screened differentially expressed proteins, and the differences in protein expression levels were displayed by volcano plot. The gene ontology (GO), Kyoto Encyclopedia of Genes and Genomes (KEGG), and Reactome pathway enrichment analysis between groups were conducted using the Majorbio Cloud platform (cloud.majorbio.com).

### 2.12 Quantitative real-time polymerase chain reaction (RT-qPCR)

The samples were frozen and ground in liquid nitrogen. The total RNA was extracted by Trizol regent (AG, China) and then reverse-transcribed into complementary DNA (cDNA) for RT-qPCR. The primer sequences for target genes and the housekeeping gene glyceraldehyde-3-phosphate dehydrogenase (GAPDH) in this study were presented in Table S1. To analyze gene expression, threshold cycle (Ct) values were obtained by processing using the 2^−^^ΔΔCT^ method.

### 2.13 Statistical analysis

All the data were presented as mean ± standard deviation (SD). Data comparisons were performed using a student's t-test between two groups or one-way ANOVA followed by Tukey's post hoc test in three or more groups via GraphPad Prism software (version 10). The significance level was indicated as *p* < 0.05 (∗), *p* < 0.01 (∗∗), and *p* < 0.001 (∗∗∗).

## 3 Results and discussion

### 3.1 Characterization of PiezoCol and NCol scaffolds

**Fig. 2:**
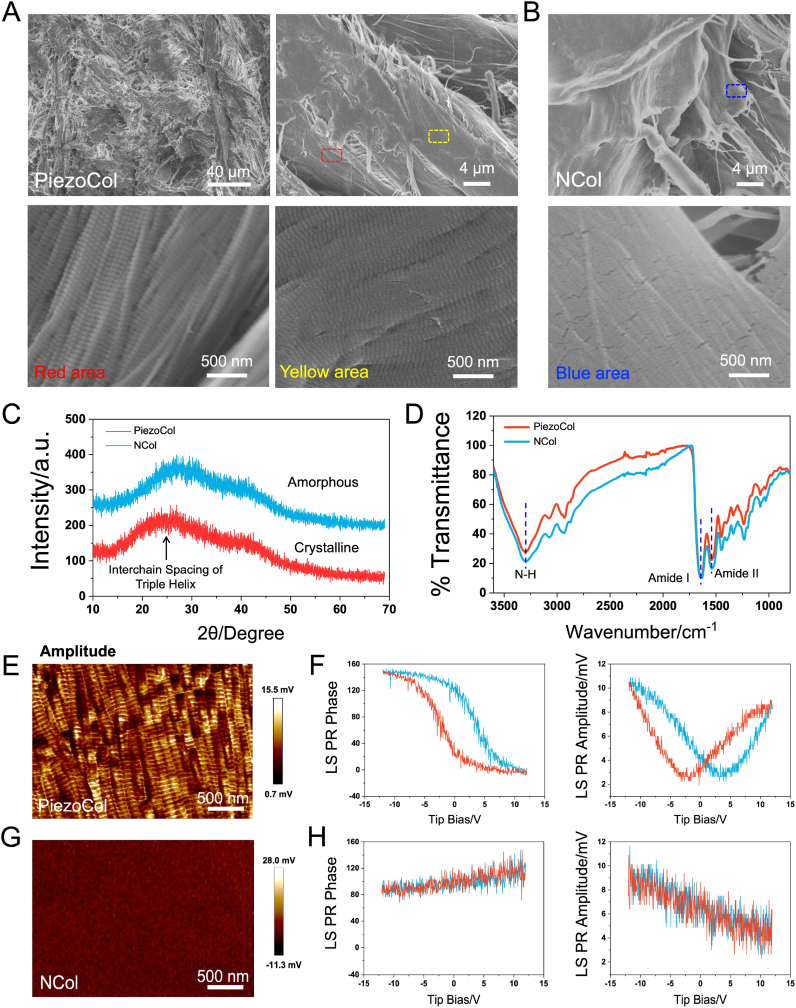
Characterization of PiezoCol and NCol scaffolds. (A) SEM images of the PiezoCol scaffold at different magnifications (red area, the inside collagen fiber structure; yellow area, the surface collagen fiber structure); (B) SEM image of the NCol scaffold at different magnifications; (C) XRD patterns of PiezoCol and NCol scaffolds; (D) FTIR spectra of PiezoCol and NCol scaffolds; (E) Amplitude map of the PiezoCol scaffold; (F) Ferroelectric phase curves and amplitude curves of the PiezoCol scaffold; (G) Amplitude map of the NCol scaffold; (H) Ferroelectric phase curves and amplitude curves of the NCol scaffold.

According to the modified process, the bioactive PiezoCol scaffold with intrinsic piezoelectricity and the denatured NCol scaffold without piezoelectricity were prepared (Fig. S1). As SEM images shown in Fig. 2A, the PiezoCol scaffold was composed of a lot of collagen fibers. To observe the detailed structure of collagen fiber, the inside (red area) and surface (yellow area) area were magnified, respectively. Both areas exhibited the typical periodic variation substructures, while the sizes of periodic variation substructures in the inside and surface area were slightly different [12]. However, after strong alkali treatment, the filamentous structure and the periodic variation substructures of natural collagen were destroyed (Fig. 2B). Furthermore, XRD results indicated that the PiezoCol scaffold possessed the characteristic inter-chain spacing peak (∼24°) of native collagen triple helix (Fig. 2C), corresponding to the previously reported result [14]. However, the peak almost disappeared after strong alkali treatment, which indicated that the complete tertiary structure of collagen was destroyed. FTIR spectra demonstrated that PiezoCol and NCol scaffolds possessed the same vibration peaks, indicating that they kept similar functional groups (Fig. 2D). However, the intensity of the amide absorbance bands (amide II, ∼1533 cm^−1^) and N-H stretching band (∼3296 cm^−1^) increased, further confirming that the complete tertiary structure of collagen was destroyed and more amide groups were exposed after strong alkali treatment [15]. DSC results indicated that the annealed PiezoCol scaffold showed only one melting peak (Tm) at around 32 °C, whereas the melting peak (Tm) slightly decreased to 31 °C after denaturation (Fig. S2). The denaturation of collagen was accompanied by an endothermic peak shift to the lower temperatures from 32 to 31 °C, which corresponded to the former study [16]. Representative tensile stress-strain curves of PiezoCol and NCol scaffolds are shown in Fig. S3. The tensile strength of the PiezoCol scaffold was ∼42 MPa, which was almost two-fold compared to the NCol scaffold (∼22 MPa). The calculated elastic modulus of the PiezoCol and NCol scaffolds were around 1.01 MPa and 0.30 MPa, respectively. Over the testing period, the degradation rate of the NCol scaffold was consistently higher than that of the PiezoCol scaffold. By day 7, the degradation ratio of the NCol scaffold reached 26.2 %, whereas that of the PiezoCol was 21.1 % (Fig. S4). This difference may be attributed to the destruction of the tertiary structure in natural collagen.

The piezoelectricity of the PiezoCol and NCol scaffolds was measured by PFM. For the PiezoCol scaffold, periodic piezoelectric signals appeared, corresponding to the morphology and structure of natural collagen fibers (Fig. 2E). Meanwhile, the standard ferroelectric butterfly amplitude curve and phase curve were obtained by applying a ramp voltage loop from −12 to 12 V (Fig. 2F). The butterfly amplitude curves showed the obvious variation by changing strain under an external field. Furthermore, the domain phase had a conspicuous switching by reversing the external electric field, which further confirmed the piezoelectric feature of the PiezoCol. The results were consistent with the previous report of collagen fibrils [17]. However, the NCol scaffold showed almost no piezoelectric signal (Fig. 2G). The standard ferroelectric butterfly amplitude curves and phase curves also could not be observed under the same conditions as well (Fig. 2H). In addition, the piezoelectric coefficients of PiezoCol and NCol scaffolds were 2.9 pC/N and 0.1 pC/N, respectively, which was consistent with the previous report [18]. Therefore, the complete tertiary structure is the key to collagen with intrinsic piezoelectricity. Interestingly, the piezoelectricity of the commercial collagen scaffold (Cossenbiot, China) used in clinic was also measured by PFM, which showed no piezoelectric signal (Fig. S6).

### 3.2 The cytocompatibility of PiezoCol and NCol scaffolds

**Fig. 3:**
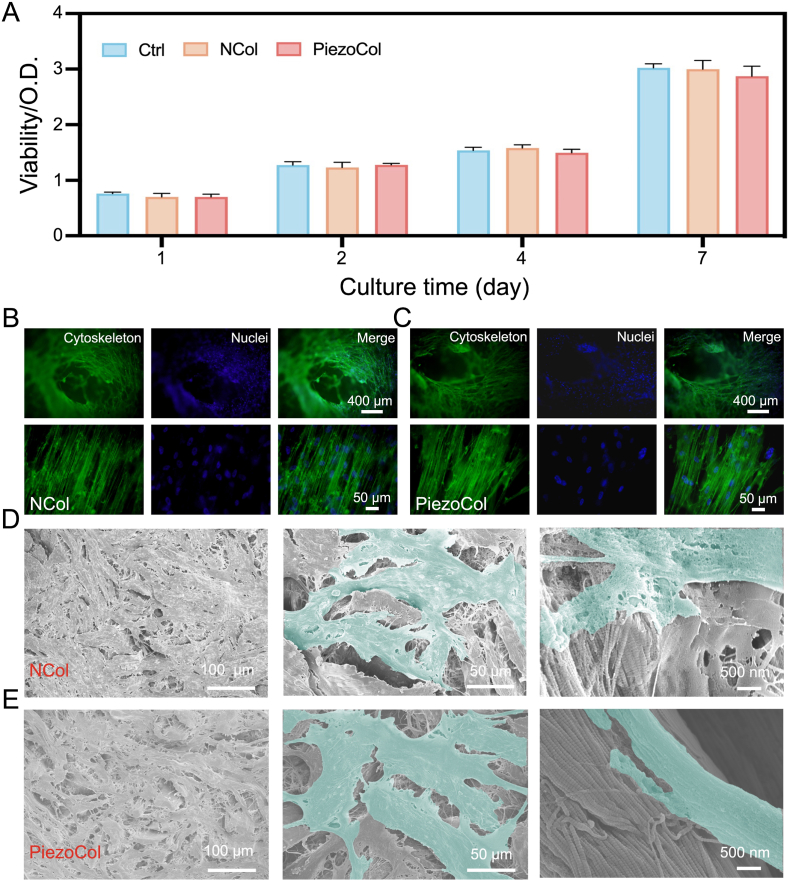
The cytocompatibility of PiezoCol and NCol scaffolds *in vitro*. (A) BMSCs viability in different groups; (B, C) Cell nuclei and cytoskeleton staining; (D, E) SEM images of BMSCs morphologies.

The cytocompatibility of PiezoCol and NCol scaffolds was measured by the CCK-8 assay kit. After 7 days of culture, BMSCs and PC12 cells cultured on both collagen scaffolds showed high viability, which was comparable with the control group (Fig. 3A and Fig. S5). The cytoskeleton and nuclei staining results confirmed that a large number of BMSCs with the typical spindle shape grew on the surface of two scaffolds, even entering the hair holes (Fig. 3B and C). The adhesion state was further assessed by SEM, and BMSCs spread well and were attached firmly on both surfaces by a lot of pseudopodia (Fig. 3D and E). All the results demonstrated that strong alkali treatment had no effect on the cytocompatibility of natural collagen, and PiezoCol and NCol scaffolds both have high cytocompatibility.

### 3.3 PiezoCol scaffold-mediated piezoelectricity for enhanced ectopic osteogenesis

**Fig. 4:**
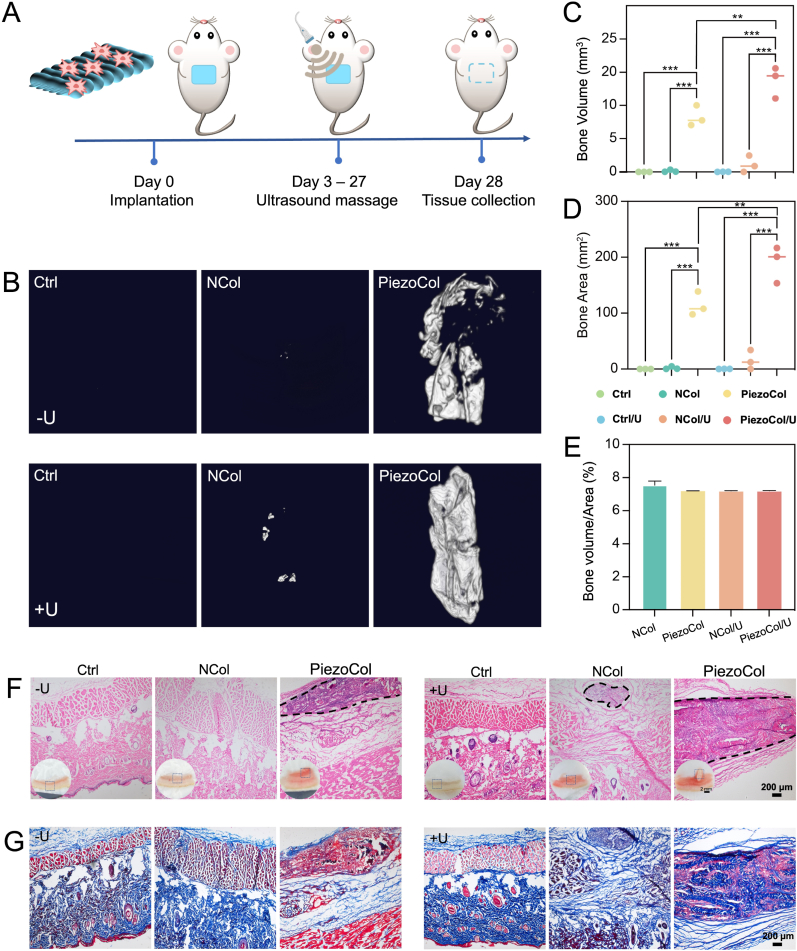
Radiologic and histologic analysis of osteogenic activities during ectopic bone formation. (A) Schematic of the fabrication of BMSCs-laden collagen scaffold and subsequent subcutaneous implantation; (B) Micro-CT images of the newly formed bone-like tissues; (C) Semi-quantitative analysis of bone volume; (D) Bone area; (E) Value of bone volume/area; (F) H&E staining of the newly formed bone-like tissue (the black lines indicate the bone-like tissue formation areas); (G) Masson staining of newly formed bone-like tissue. ∗∗*p* < 0.01, ∗∗∗*p* < 0.005.

The biological effects of PiezoCol scaffold-mediated piezoelectricity on osteogenesis were subsequently investigated *in vivo* using an ectopic bone formation rat model. The two kinds of BMSCs-laden collagen scaffolds were implanted subcutaneously on the dorsal part of rats. To study the effect of piezoelectricity on osteogenesis, ultrasound treatment was performed every 2 days until the surrounding tissues were collected for further analysis (Fig. 4A). The specimens were collected at 4 weeks after implantation for radiological and histological analysis. Micro-CT images demonstrated that no new bone was formed in the control groups with/without ultrasound treatment, even though BMSCs were injected subcutaneously (Fig. 4B). Similarly, ultrasound treatment had a scarce effect on bone-like tissue formation in NCol groups. In contrast to NCol groups, robust bone-like tissue formation was observed in PiezoCol groups, particularly after ultrasound treatment. Semi-quantitative analysis showed that the bone volume and bone area were significantly increased up to around twofold when ultrasound treatment were performed in the PiezoCol group (*p* < 0.01) (Fig. 4C and D). We speculated that the normal movement of rats could produce piezoelectricity, whereas the efficiency was low. The ultrasound treatment enhanced the promotion of piezoelectricity-induced osteogenesis. The value of bone volume/area was calculated to evaluate the bone-like tissue quality in different groups (Fig. 4E). Further, there was no obvious difference among each group in the values of bone volume/area, confirming that the ultrasound treatment supported new bone formation without compromising bone quality.

Histological analysis also revealed more bone-like tissue formation as well as more active osteogenesis in PiezoCol groups than in other groups. The sections around bone-like tissue were studied through H&E and Masson staining. H&E staining showed that collagen scaffolds almost degraded after 4 weeks. A significant amount of bone-like tissue was observed in both PiezoCol groups, especially after ultrasound treatment (Fig. 4F). Interestingly, within the newly formed tissue, cells distributed densely, and the amount was proportional to the tissue volume, which might indicate an active bone formation process. Masson staining showed a similar trend as H&E staining (Fig. 4G). Compared with NCol groups, the newly formed bone-like tissue in PiezoCol groups showed a higher level of collagen matrix maturation, demonstrating the beneficial effect of piezoelectricity on hard tissue development.

**Fig. 5:**
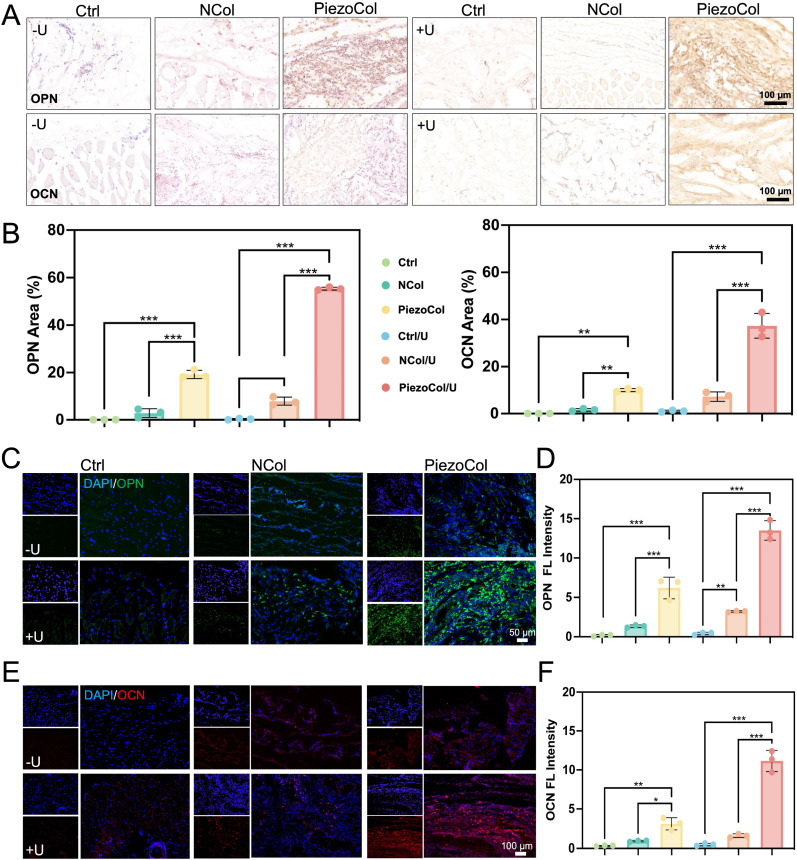
Expression of osteogenesis-related markers *in vivo*. (A) IHC staining of OPN and OCN in the area around newly formed bone-like tissue; (B) Semi-quantitative analysis of IHC staining of OPN and OCN; (C) IF staining for OPN; (D) Semi-quantitative calculation of fluorescence density for OPN; (E) IF staining for OCN; (F) Semi-quantitative calculation of fluorescence density for OCN. ∗*p* < 0.05, ∗∗*p* < 0.01, ∗∗∗*p* < 0.005.

In addition, IHC staining was performed to examine the osteogenic differentiation at the new bone-like tissue formation areas. For osteogenic markers, OPN and OCN were chosen, which represented the relatively later stage of osteogenic differentiation [19]. In Ctrl and NCol groups without ultrasound treatment, no obvious protein was expressed. The NCol group that underwent ultrasound treatment showed a scarce amount of protein expression. Nevertheless, both PiezoCol groups showed a significantly elevated protein expression, and ultrasound treatment showed the highest promotion (Fig. 5A). Later, the semi-quantification of OPN and OCN expression was calculated. Results from both Ctrl groups indicated that alone ultrasound treatment showed no effect on BMSCs osteogenic differentiation without scaffolds. Interestingly, the OPN and OCN expression was a little bit higher in the NCol/U group than that without ultrasound treatment. Those osteogenic protein expressions might be attributed to the mechanical vibration caused by ultrasound [20]. The OPN and OCN expression in the PiezoCol group was almost 7 times than those in the NCol group without ultrasound treatment, which might be due to the movement-induced piezoelectric stimulation. In the PiezoCol/U group, OPN and OCN expression was elevated significantly compared with the non-ultrasound treatment group, indicating the positive role of ultrasound treatment when applying the PiezoCol scaffold (Fig. 5B).

IF staining was performed to further confirm the osteogenesis among different groups. Corresponded to IHC results, in groups without scaffolds, ultrasound treatment showed no effect on BMSCs osteogenic differentiation. In NCol groups, ultrasound treatment elevated osteogenesis slightly, which might be due to mechanical stimulation on BMSCs differentiation, as we discussed above. Similar to the IHC analysis, PiezoCol groups, either with or without ultrasound treatment, showed efficient OPN expression, whereas the PiezoCol group with ultrasound treatment showed the highest level of fluorescent intensity, indicating better osteogenic capacity after piezoelectricity stimulation (Fig. 5C and D). The OCN expression showed a similar trend to the OPN expression, demonstrating the strongest expression in the PiezoCol/U group (Fig. 5E and F).

We further explored the osteoclast activity of bone-like tissue formation areas in the PiezoCol/U group by TRAP staining. Osteoclasts were actively distributed around the newly formed bone-like area in the PiezoCol group after ultrasound treatment, whereas no obvious TRAP-positive cells were found in the control group (Fig. S7). Generally, in the new bone formation area, more osteoclast distribution means active bone remodeling, indicating a more active osteogenic process in the PiezoCol/U group.

**Fig. 6:**
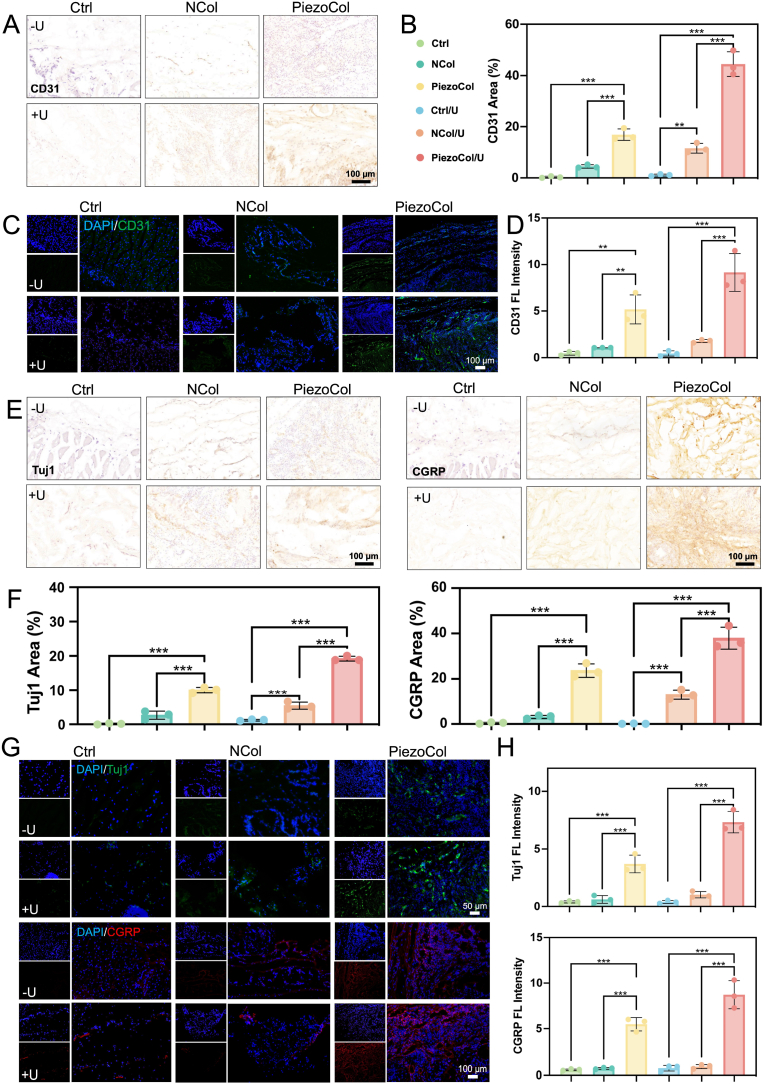
Expression of angiogenesis and neurogenesis-related markers *in vivo*. (A) IHC staining of CD31; (B) Semi-quantitative analysis of IHC staining of CD31; (C) IF staining for CD31; (D) Semi-quantitative calculation of fluorescence density for CD31; (E) IHC staining of Tuj1 and CGRP in the area around newly formed bone-like tissue; (F) Semi-quantitative analysis of IHC staining of Tuj1 and CGRP; (G) IF staining for Tuj1 and CGRP; (H) Semi-quantitative calculation of fluorescence density for Tuj1 and CGRP. ∗∗*p* < 0.01, ∗∗∗*p* < 0.005.

To achieve satisfactory new bone formation and function reconstruction, ideal bone repair scaffolds should not only improve osteogenesis but also promote blood vessel and nerve formation [21]. Subsequently, we performed CD31 IHC and IF staining to observe neovascularization during the new bone formation phase. Results indicated that no CD31 was expressed without scaffolds, even though ultrasound treatment was applied. However, in NCol groups, the CD31 expression was slightly increased, which might be attributed to the support provided by scaffolds for the recruitment and proliferation of endothelial cells [22]. Both IHC and IF staining results showed that the PiezoCol group with ultrasound treatment exhibited the strongest CD31 expression, followed by the PiezoCol group without ultrasound treatment. Specifically, compared to NCol groups, the expressions of CD31 in PiezoCol groups were around 3-fold higher (Fig. 6A–D). Based on these findings, it is reasonable to speculate that the piezoelectric stimulation via the PiezoCol scaffold promoted angiogenesis, which further influences osteogenesis-angiogenesis coupling in bone regeneration.

As bone is a highly innervated tissue, the role of bone-nerve crosstalk in bone repair is also critical beyond osteogenesis-angiogenesis coupling [23]. Therefore, the IHC and IF staining of neurogenesis-related markers Tuj1 and CGRP were conducted. The results showed that both Tuj1 and CGRP expression were significantly increased in the PiezoCol groups (Fig. 6E–H), indicating that the PiezoCol scaffold displayed a promoting effect on neurogenesis during bone regeneration. Particularly, compared with the non-treatment group, the neurogenic activity was enhanced after ultrasound treatment in the PiezoCol/U group which showed the highest Tuj1 and CGRP expression. These findings also demonstrated the supportive role of piezoelectricity in nerve formation during bone regeneration.

In addition, the biocompatibility and biosecurity of BMSCs-laden collagen scaffolds were assessed *in vivo*. There were no obvious changes in the major organs, including heart, liver, spleen, lung, and kidney tissues in the scaffold groups compared with the control group, either with or without ultrasound treatment from the results of H&E staining (Fig. S8). Moreover, the blood analysis demonstrated that the routine blood indices were similar among those three groups, confirming the good biosecurity of the collagen scaffolds (Fig. S9).

### 3.4 The mechanism of piezoelectric effect on osteogenesis during bone formation

**Fig. 7:**
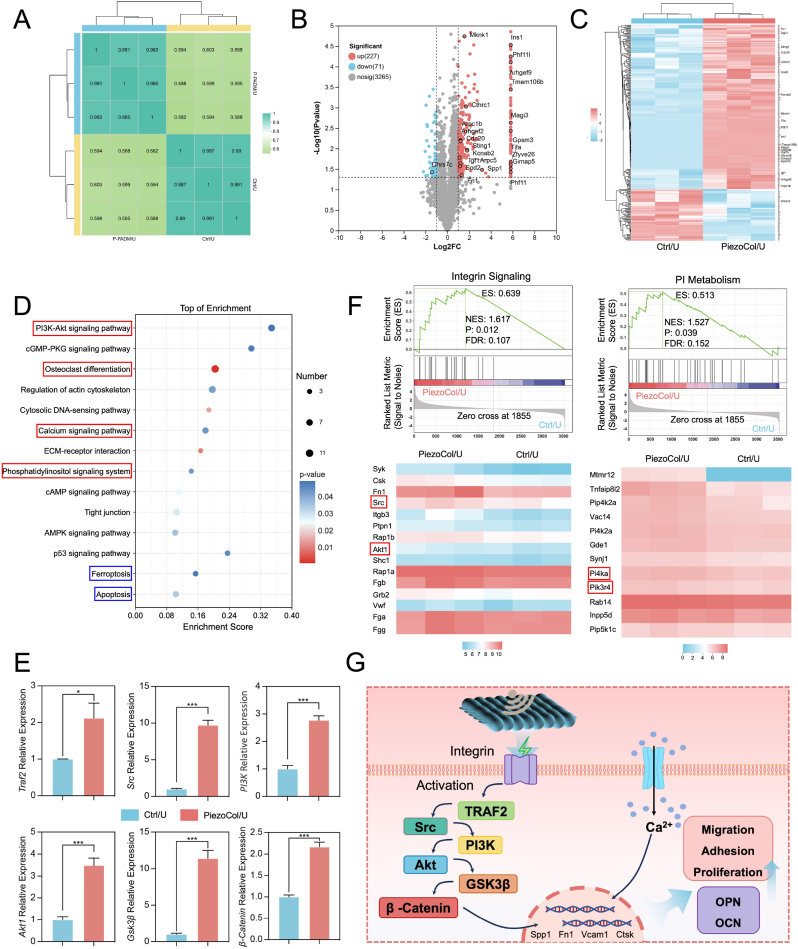
Proteomic study on the mechanism of piezoelectric effect on bone formation. (A) Heat map of sample-to-sample distances; (B) Volcano plot of proteomic analysis of the differential proteins between the Ctrl/U and PiezoCol/U groups; (C) Heat map of the differential proteins between the Ctrl/U and PiezoCol U groups; (D) Bubble map of the KEGG enrichment analysis of osteogenic signaling pathways; (E) Expression of key genes in PI3K-Akt signaling pathway; (F) GSEA and leading edge in phosphatidylinositol metabolism and integrin signaling; (G) The comprehensive schematic of the mechanism of bone formation via piezoelectric stimulation. ∗*p* < 0.05, ∗∗∗*p* < 0.005.

To further explore the mechanism of piezoelectricity supporting osteogenesis, a label-free quantitative proteomic analysis with the newly formed bone-like tissue was performed; three independent experimental specimens in each group were consistent (Fig. 7A). DEPs were selected according to the threshold value of |log2FC| ≥ 1 and *p*-value < 0.05. According to the results of the volcano plot and heat map, 298 differential proteins have been found, of which 227 proteins are up-regulated and 71 proteins are down-regulated (Fig. 7B and C). The top 20 up-regulated proteins were highlighted in the heat map. Those up-regulated proteins, such as FN1 and SPP1, are reported to be related to osteogenesis [24,25]. Results of the GO annotations analysis indicated that the up-regulated proteins, including biological process, cellular component, and molecular function were more than those down-regulated. Some proteins are possibly associated with piezoelectricity-promoted osteogenesis, such as response to stimulus and locomotion (Fig. S10). Moreover, GO functional enrichment analysis showed several up-regulated and down-regulated proteins that might be associated with osteogenesis, such as positive regulation of cell activation, regulation of cytokine production, regulation of mononuclear cell proliferation, and regulation of cell activation (Fig. S11).

Subsequently, the differential protein expression was analyzed using the KEGG pathway enrichment analysis (Fig. 7D). The results of relevant top-enriched up-KEGG pathways indicated that several up-regulated pathways were related to osteogenic activities. Among those pathways, the phosphoinositide 3-kinase (PI3K)-protein kinase B (Akt) signaling pathway was upregulated with a higher enrichment score. PI3Ks are a family of enzymes participating in various cellular functions and metabolic activities, including cell growth, proliferation, differentiation, survival, intracellular trafficking, etc. PI3K-Akt pathway is one of the most typical downstream signaling pathways following the extracellular matrix-integrin interaction in osteoblasts [26]. Phosphoinositides are generated following the activation of PI3K via insulin or growth factor stimulation, which is crucial for the membrane targeting of pleckstrin homology domain-containing proteins such as Akt [27]. To confirm the positive role of up-regulated PI3K-Akt pathway in piezoelectricity-promoted osteogenesis, we picked up and studied the expression of key genes located in this pathway, including *Traf2*, *Src, PI3K*, *Akt1*, *Gsk3*β, and *β-Catenin* [28,29]. Results showed that all the related gene expressions were significantly up-regulated with piezoelectric stimulation (Fig. 7E), indicating the critical role of the PI3K-Akt signaling pathway in piezoelectricity-promoted osteogenesis. Previous studies have reported that up-regulating the PI3K/Akt/GSK3β/β-catenin signaling pathway could promote osteoblast maturation and mineralization [30,31]. Notably, piezoelectric stimulation could enhance tissue regeneration by activating the PI3K-Akt signaling pathway [32]. Therefore, it could be inferred that the osteogenesis of BMSCs resulted from the activation of the PI3K/Akt/GSK3β/β-catenin signaling pathway with piezoelectric stimulation. In addition, this signaling pathway has been widely accepted to participate in the regulation of the BMSCs migration and recruitment and then to regulate the synthesis of osteogenesis-related proteins, such as OPN and OCN [[33], [34], [35]].

The calcium signaling pathway is another targeted signaling pathway participating in regulating intracellular Ca^2+^ concentration and driving osteogenesis after activation. The changes in intracellular Ca^2+^ concentration, termed Ca^2+^ oscillations, play an important role in osteoblast differentiation by influencing cellular processes through the activation of specific signaling pathways like the Ca^2+^/Calcineurin/NFAT signaling pathway and the response to mechanical stimuli [36,37]. More importantly, the former study suggested that stem cells showed slow and spontaneous Ca^2+^ oscillations, and regulating Ca^2+^ signaling by electrical stimulation could promote their differentiation *in vivo* [38]. Therefore, we stepped back and measured the intracellular Ca^2+^ content *in vitro*. Results showed that piezoelectric stimulation increased intracellular Ca^2+^ content during osteogenic differentiation, and the mechanical vibration caused by ultrasound almost had no effect on intracellular Ca^2+^ content (Fig. S12). PiezoCol-mediated piezoelectric signal with the assistance of ultrasound can promote Ca^2+^ influx, leading to the content of intracellular Ca^2+^ increase and Ca^2+^ oscillations. The increased Ca^2+^ content promoted osteogenic differentiation. Inversely, NCol cannot provide piezoelectric stimulation, failing to accelerate cell differentiation. In correspondence to our results, previous studies also reported that piezoelectric stimulation could enhance osteogenic activity via regulating calcium signaling [39,40]. Moreover, the osteoclast signaling pathway was upregulated, which corresponded to the TRAP staining result, confirming the active osteogenic activity. The KEGG enrichment analysis also revealed several down-regulated pathways, such as apoptosis and ferroptosis, which were usually thought to be beneficial to tissue regeneration [24,41].

Furthermore, gene set enrichment analysis (GSEA) indicated that various osteogenesis-related biological processes were up-regulated, including integrin signaling and phosphatidylinositol (PI) metabolism (Fig. 7F). The integrin signaling pathway plays a critical role in osteogenesis [42]. Previous studies indicated that integrin protein (ITG) as the key factors, especially ITGA3, ITGB1, ITGB3, and ITGB4, interact with PI3K and Akt. The PI3K linked to Akt can be activated by ITG subsequently [42,43], which is confirmed in the current study. PI metabolism is a crucial part of intracellular signal transduction, involving a variety of biochemical processes [44]. PI is a type of phospholipid that is a major component of cell membranes and is the precursor to phosphoinositides. Phosphoinositides are formed when one or more phosphate groups are added to the inositol ring of PI at different positions (3, 4, or 5) [45]. In the leading edge of PI metabolism, Pik3r4 is a component of the PI3K-Akt pathway, which was reported to play an important role in promoting bone formation [46]. Moreover, another enriched protein Pi4ka is a kinase participating in phosphoinositide synthesis, which might relate to cellular signal conduction and cell differentiation [47]. Based on the above results, it could be inferred that the osteogenesis of BMSCs-laden on PiezoCol resulted from the activation of the integrin/PI3K/Akt signaling pathway by piezoelectricity. In the meantime, the calcium signaling pathway participated in osteogenesis via Ca^2+^ ossification. Therefore, the piezoelectricity may promote osteogenic differentiation of BMSCs via activating the integrin/PI3K/Akt signaling axis and regulating the calcium signaling pathway (Fig. 7G).

**Fig. 8:**
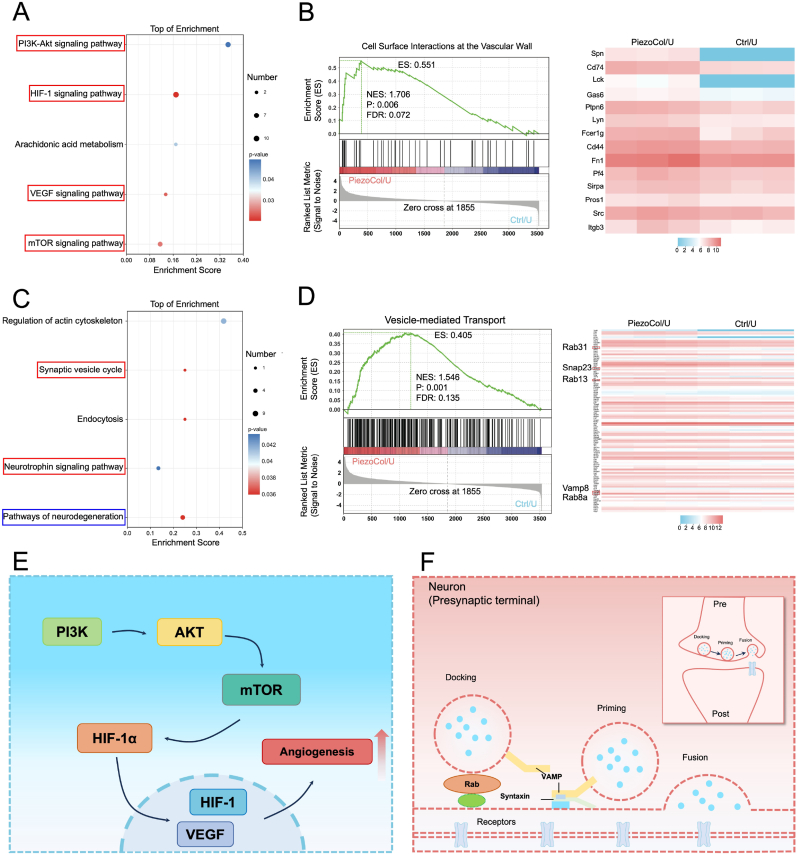
Proteomic study on the mechanism of piezoelectric effect on angiogenesis and neurogenesis. (A) Bubble map of the KEGG enrichment analysis of angiogenic signaling pathways; (B) GSEA and leading edge in cell surface interactions at the vascular wall. (C) Bubble map of the KEGG enrichment analysis of neurogenic signaling pathways; (D) GSEA and leading edge in vesicle-mediated transport; (E) The schematic of the mechanism of angiogenesis via piezoelectric stimulation. (F) The schematic of the mechanism of neurogenesis via piezoelectric stimulation.

Additionally, the KEGG pathway enrichment analysis indicated that piezoelectric stimulation enhanced angiogenesis, as relevant pathways including hypoxia-induced factor-1 (HIF-1) signaling pathway, vascular endothelial growth factor (VEGF) signaling pathway were up-regulated (Fig. 8A). Results of GSEA also confirmed that DEPs were enriched in the cell surface interactions in the vascular wall (Fig. 8B). The PI3K/Akt/mTOR signaling pathway regulated the expression of HIF-1α, and then HIF-1α further regulated the expression of downstream proteins involved in angiogenesis, such as VEGF [48]. Combined with our above results, hereby, we speculate that the piezoelectric stimulation could promote angiogenesis via the PI3K/Akt/mTOR/HIF-1 signaling pathway (Fig. 8E). Meanwhile, the top enriched KEGG pathways revealed that the stimulation from PiezoCol enhanced neurogenesis as several relevant pathways were upregulated (Fig. 8C). Particularly, the synaptic vesicle cycle with a higher enrichment score has been reported to be highly associated with neurogenesis [49]. Further evidence from GSEA indicated that the DEPs, including Rab31, Snap23, Rab13, Vamp8, and Rab8a were enriched in vesicle-mediated transport (Fig. 8D and F). Even though no direct evidence has been published between the synaptic vesicle cycle and osteogenesis, neurogenesis plays irreplaceable roles in bone regeneration and bone defect repair [50]. Therefore, more studies are required for a deep understanding of the relationship between osteogenesis and neurogenesis. Based on the above results, it could be inferred that the piezoelectric stimulation derived from the PiezoCol scaffold promoted osseointegration also by regulating the coupling of angiogenesis and neurogenesis *in vivo*.

It is well known that exercise is well-documented to benefit bone, brain, cardiovascular, and musculoskeletal health [51], and can reduce marrow adiposity, increase bone density, and promote nerve regeneration [52,53]. However, limited studies have explored how exercise influences cellular behavior and differentiation, which is key to fully understanding its health benefits at the cellular level. Exercise-induced collagen deformation can lead to the generation of piezoelectricity, which may be one of the mechanisms by which exercise promotes bone health and nerve regeneration as the PiezoCol scaffold alone also accelerates bone regeneration, providing another promising strategy for future treatment of bone defects.

Piezoelectric signals play a vital role in bone repair and growth. Piezoelectricity arises from the sliding of collagen fibers under mechanical forces, generating charges that enhance osteoblast function and promote regeneration. The endogenous electric field such as intrinsic piezoelectricity within living bone effectively regulates intracellular metabolism [54]. Therefore, to fully make use of such bioelectric signals for bone repair, biomaterial scaffolds with intrinsic piezoelectricity may be a promising strategy. Exercise-induced collagen deformation can lead to the generation of piezoelectricity, facilitating bone repair and remodeling. More importantly, collagen is the most abundant protein in the human body and has become one of the most important natural and FDA-proved biomaterials for tissue regeneration due to its excellent biocompatibility, showing the natural advantages over other piezoelectric materials such as BaTiO_3_ and PVDF. However, before clinical application, some limitations are still required to be solved. For instance, the current sizes of the PiezoCol scaffold are small due to the decellularization-based producing method, making it only applicable to small bone defects. Therefore, future studies are required to modify the process to produce PiezoCol scaffolds with tailored sizes that can fit all kinds of bone defect sites.

## 4 Conclusion

This study prepared a natural collagen scaffold with a complete tertiary structure based on the porcine acellular dermal matrix, which showed good piezoelectricity. The collagen scaffold lost piezoelectricity after alkali treatment. PiezoCol/NCol scaffolds both possessed good cytocompatibility, and stem cells can adhere and spread well on both surfaces. PiezoCol/NCol scaffolds were implanted subcutaneously to assess the *in vivo* bone regeneration. The PiezoCol scaffold promoted the new bone formation with enhanced osteogenic differentiation, angiogenesis, and neural differentiation under ultrasound treatment. Meanwhile, the PiezoCol scaffold alone can also accelerate bone formation, possibly attributed to the daily exercise-caused weak piezoelectric stimulation. Inversely, the NCol scaffold almost did not affect bone regeneration with or without ultrasound treatment. Further, the proteomic analysis revealed the mechanism by which the PiezoCol scaffold promoted bone tissue formation via mainly upregulating the PI3K-Akt signaling pathway. The calcium signaling pathway was also participated through calcium ossification. However, the prepared PiezoCol still has some limitations, such as the thickness of the scaffold, due to its source and preparation process, requiring further optimization to promote application in large bone defect areas. To overcome this obstacle, modification of the process to produce PiezoCol scaffolds with tailored sizes that can fit all kinds of bone defect sites is required. In conclusion, this research provides a new strategy to enhance the osteoinduction of collagen scaffold for bone regeneration by maintaining intrinsic piezoelectricity.

## CRediT authorship contribution statement

**Jing Han:** Writing – original draft, Methodology, Investigation. **Zhao Li:** Investigation. **Jing Du:** Validation, Methodology. **Qun Zhang:** Methodology, Investigation. **Shaohua Ge:** Writing – review & editing, Supervision, Conceptualization. **Hong Liu:** Supervision, Conceptualization. **Baojin Ma:** Writing – review & editing, Supervision, Funding acquisition, Conceptualization.

## Declaration of competing interest

The authors declare that they have no known competing financial interests or personal relationships that could have appeared to influence the work reported in this paper.

## Data Availability

Data will be made available on request.
