# Supplementary material for: Natural collagen scaffold with intrinsic piezoelectricity for enhanced bone regeneration
Source: Mater Today Bio. 2025 Jan 29;31:101532. doi: 10.1016/j.mtbio.2025.101532 (PMC11834078; doi:10.1016/j.mtbio.2025.101532)
Supplement: Multimedia component 1 [file mmc1.docx]

**Supporting information**

**Natural Collagen Scaffold with Intrinsic Piezoelectricity for Enhanced Bone Regeneration**

Jing Han ^a1^, Zhao Li ^b1^, Jing Du ^a^, Qun Zhang ^a*^, Shaohua Ge ^a*^, Hong Liu ^c^, Baojin Ma ^a*^

^a^ Department of Tissue Engineering & Periodontology, School and Hospital of Stomatology, Shandong University & Shandong Key Laboratory of Oral Tissue Regeneration & Shandong

Engineering Research Center of Dental Materials and Oral Tissue Regeneration & Shandong Provincial Clinical Research Center for Oral Diseases, Jinan, Shandong, 250012, China

^b^ College of Materials Science and Engineering, Qingdao University of Science & Technology, Qingdao, Shandong, 266061, China

^c^ State Key Laboratory of Crystal Materials, Shandong University, Jinan, Shandong, 250013, China

E-mail: [zhangqunpku@126.com](mailto:zhangqunpku@126.com) (Q. Zhang); [shaohuage@sdu.edu.cn](mailto:shaohuage@sdu.edu.cn) (S. Ge); [baojinma@sdu.edu.cn](mailto:baojinma@sdu.edu.cn) (B. Ma)


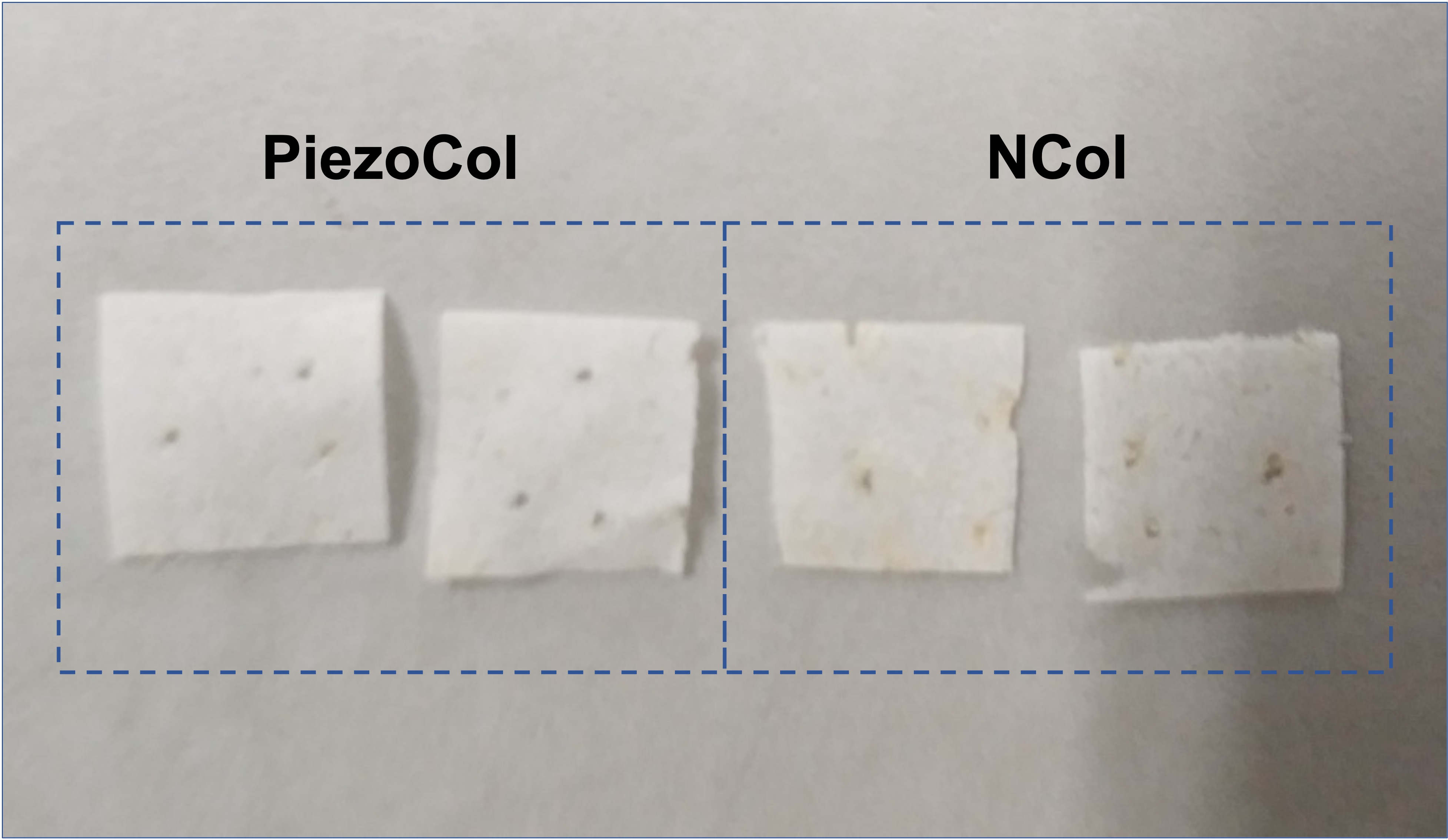


Fig. S1. Digital picture of P-PADM and N-PADM.


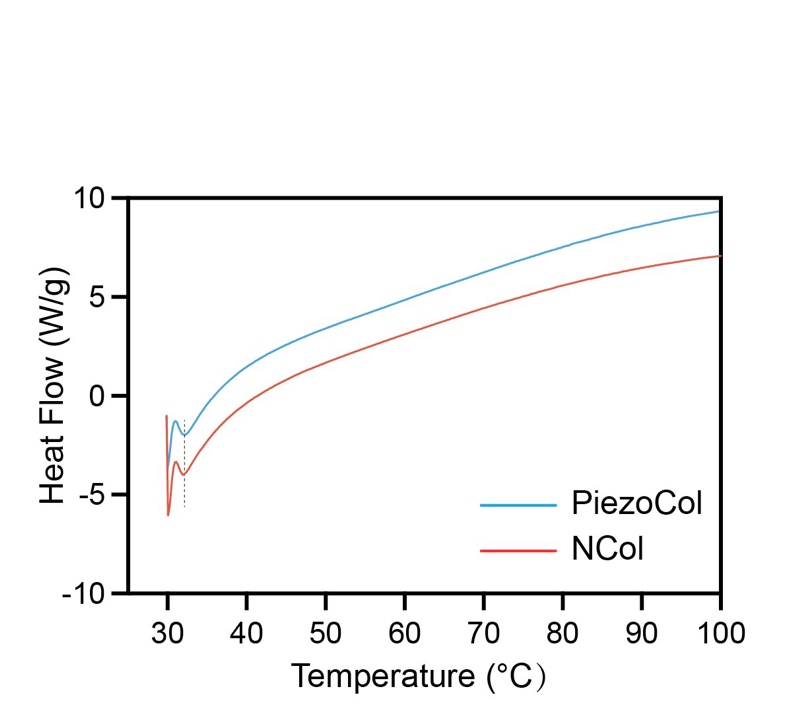


Fig. S2. DSC curves of PiezoCol and NCol scaffolds.


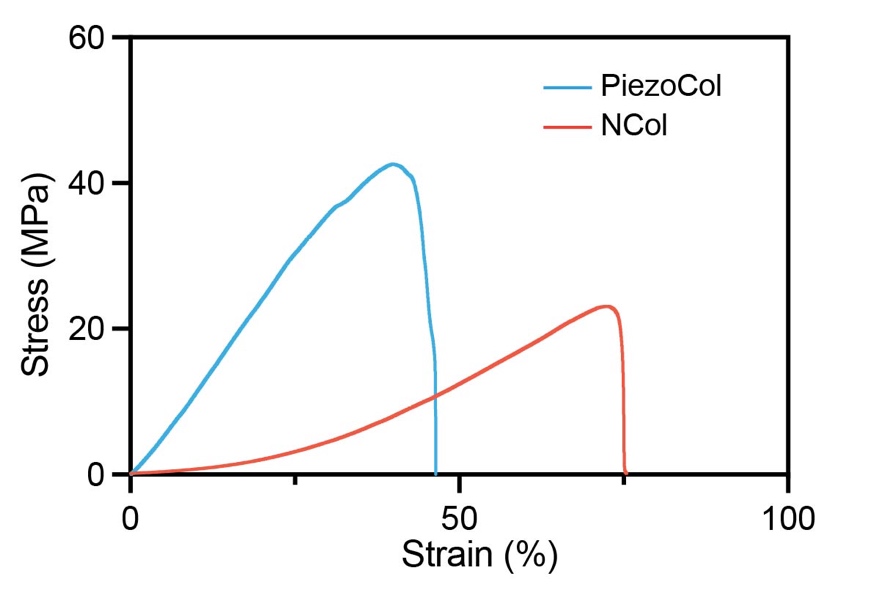


Fig. S3. Stress-strain curve of PiezoCol and NCol scaffold.


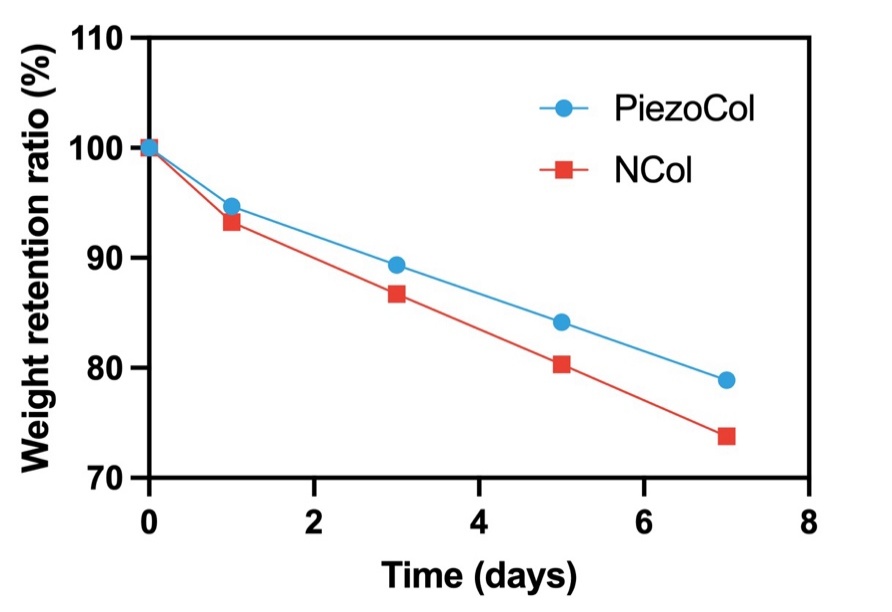


Fig. S4. Retention ratio of PiezoCol and NCol scaffold *in vitro* after immersion in PBS with collagenase at the predetermined time intervals.


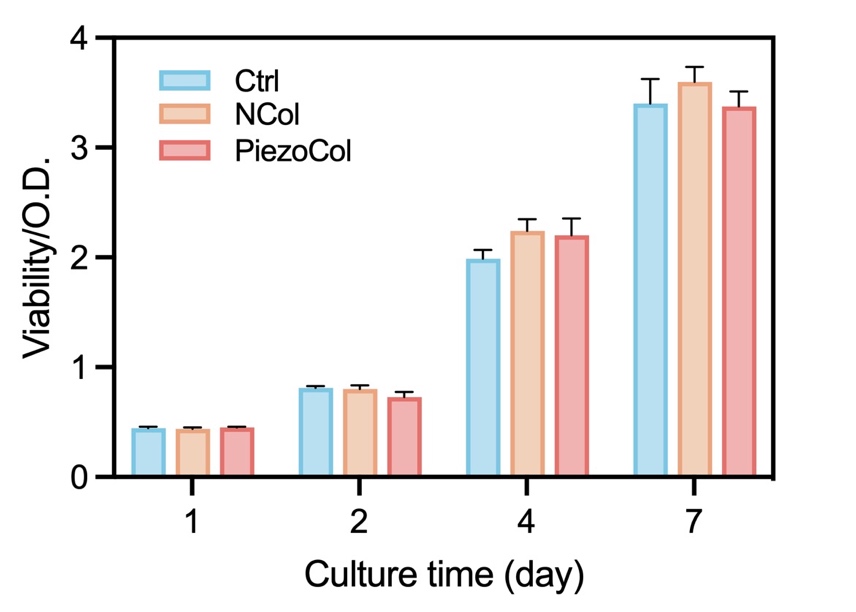


Fig. S5. PC12 cell viability in different groups.


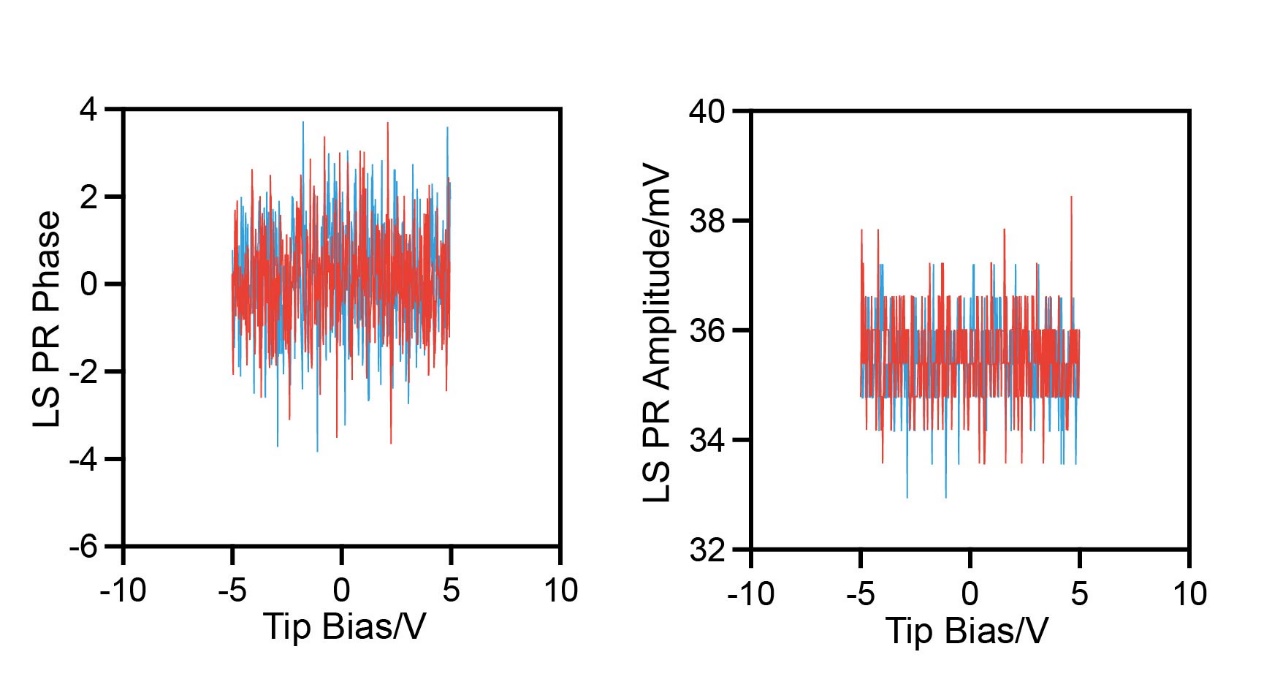


Fig. S6. Ferroelectric phase curves and amplitude curves of the commercial collagen scaffold


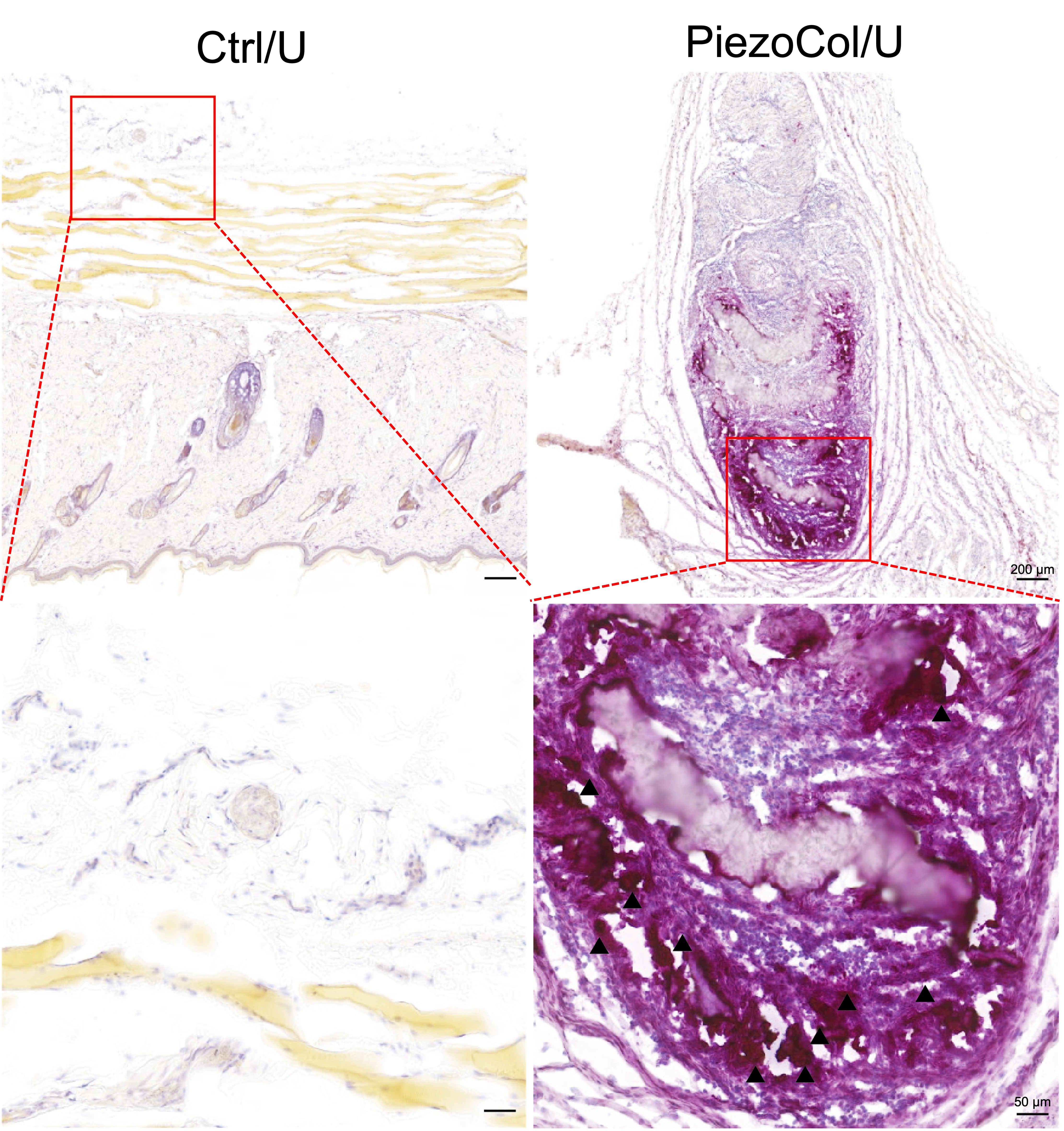


Fig. S7. TRAP staining of cells around bone-like tissue formation area (black triangles indicate the osteoclasts).


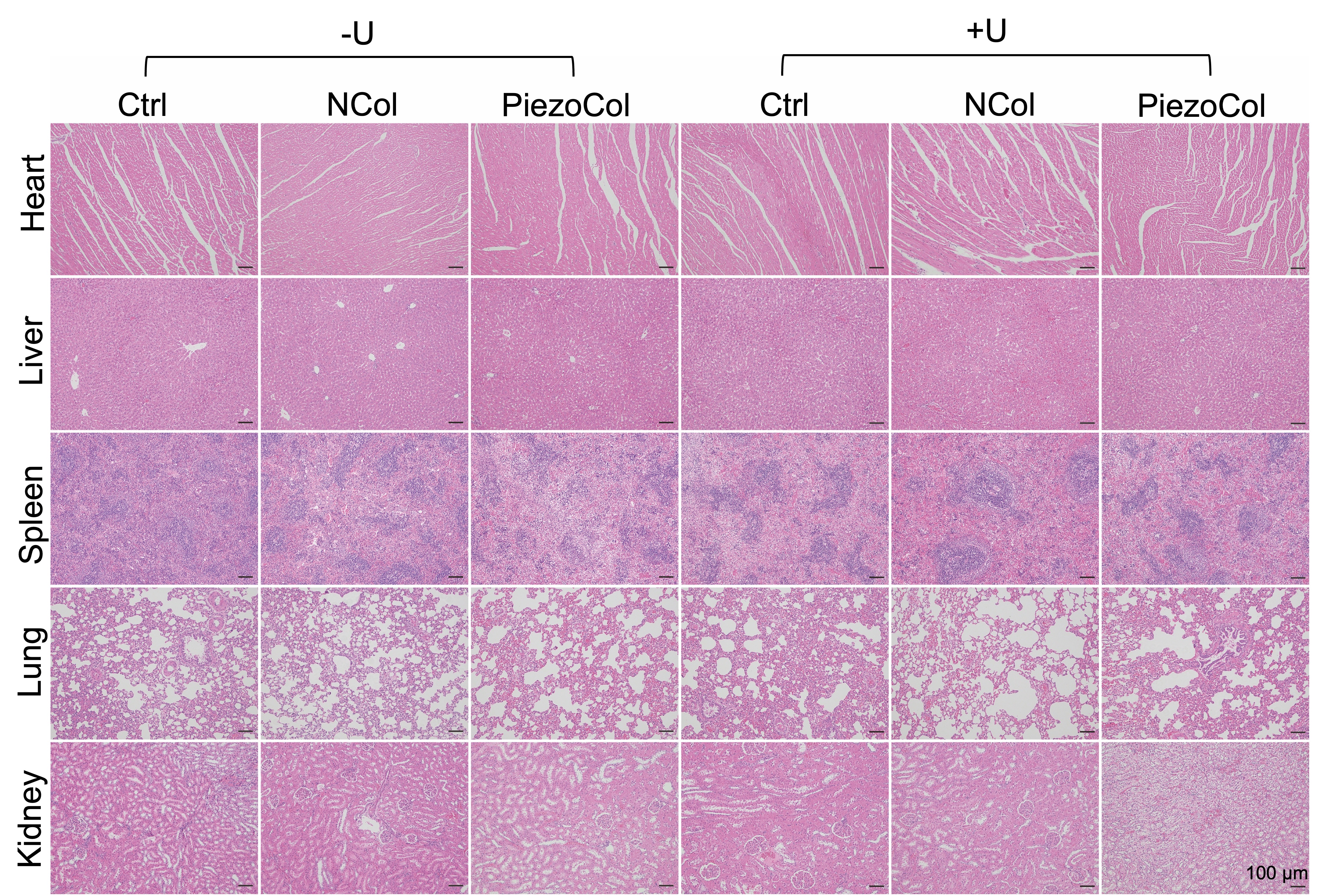


Fig. S8. Biocompatibility assessment *in vivo*. The H&E staining of tissue slices in the heart, liver, spleen, lung, and kidney.


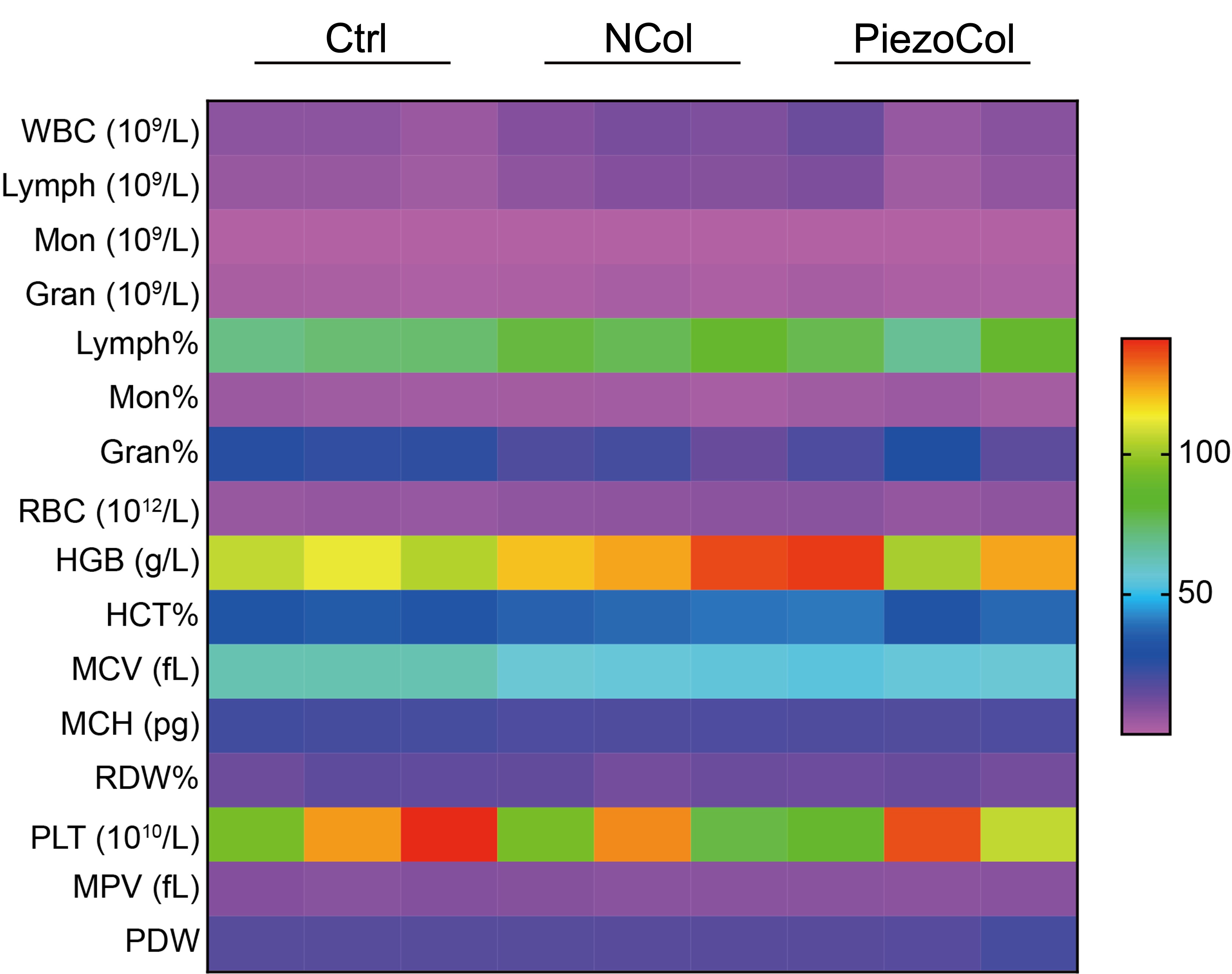


Fig. S9. The routine blood indices of rats after different treatment, including white blood cell count (WBC), lymphocyte count (Lymph), monocyte count (Mon), granulocyte count (Gran), lymphocyte percentage (Lymph%), monocyte percentage (Mon%), granulocyte percentage (Gran%), red blood cell count (RBC), hemoglobin (HGB), hematocrit (HCT), mean corpuscular volume (MCV), mean corpuscular hemoglobin (MCH), (RDW%), platelet count (PLT), mean platelet volume (MPV), platelet distribution width (PDW).


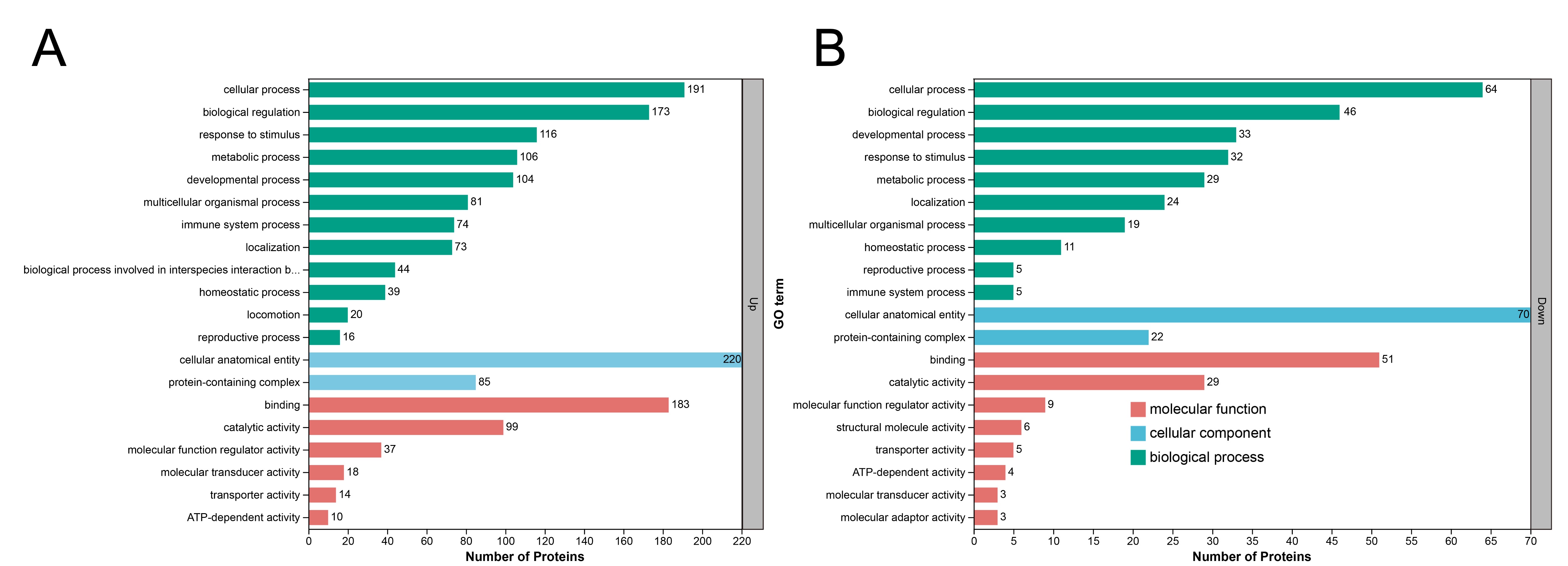


Fig. S10. GO annotation analysis of PiezoCol/U vs. Ctrl/U.


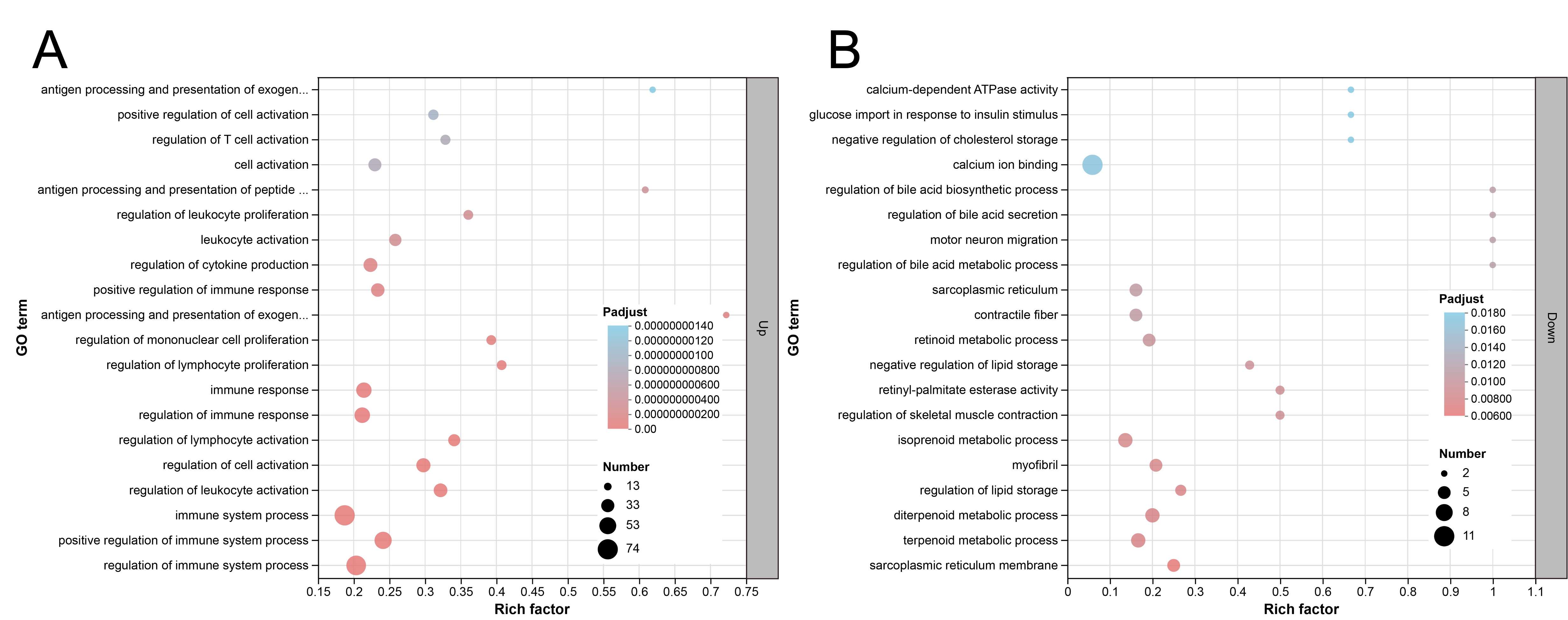


Fig. S11. GO enrichment analysis of PiezoCol/U vs. Ctrl/U.


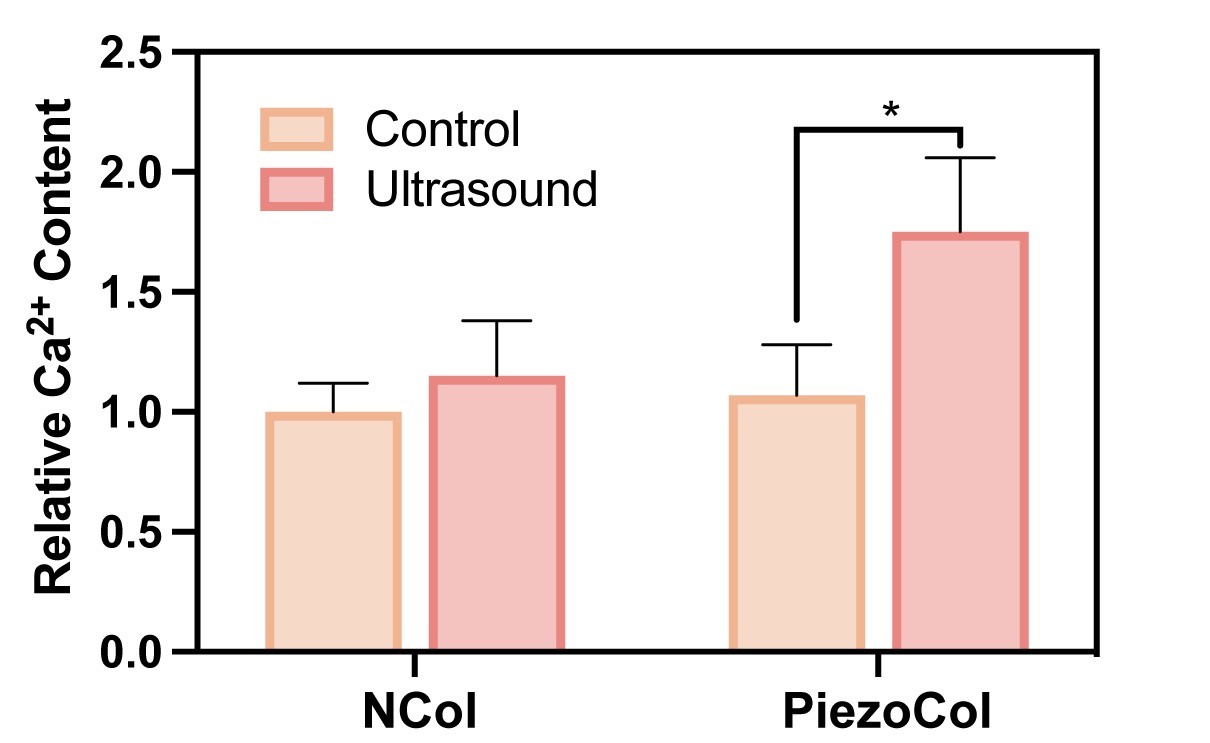


Fig. S12. Piezoelectric stimulation mediated Ca^2+^ influx. The content change of Ca^2+^ in cells with/without piezoelectricity stimulation during osteogenic differentiation.

**Table S1.** Osteogenic-related gene primer sequences used in the RT-qPCR experiments.

| Gene Name | Forward Sequences | Reverse Sequences |
| --- | --- | --- |
| Traf2 | TCCGACTTCGCCAGAAAACG | GGGACCTTTCATCACCACGA |
| Src | TGTGTCACCGTCTCACTACCG | GGCTTCAGGGTTTTGATGGC |
| PI3k | TACTGCGTGGCAACCTTTATC | GCTGTCCGTCATCTTTCACCA |
| Akt1 | TACGGTGCGGAGATTGTGTC | ACAGCCCGAAGTCCGTTATC |
| Gsk3β | TCCGAGGAGAGCCCAATGTT | CGTGTAATCGGTGGCTCCAA |
| β-Catenin | CTCGGACTGGACATTGGTGC | ATAGGGTCCATCCCCAAGGC |
| GAPDH | TCTCTGCTCCTCCCTGTTCT | ATCCGTTCACACCGACCTTC |
